# Serum titin/creatinine ratio as a biomarker for discriminating disease severity in Duchenne and Becker muscular dystrophies

**DOI:** 10.3389/fneur.2025.1591748

**Published:** 2025-07-09

**Authors:** Yoshinori Nambu, Kayo Osawa, Taku Shirakawa, Aiko Sunami, Shoko Sonehara, Ryosuke Bo, Kandai Nozu, Masafumi Matsuo, Hiroyuki Awano

**Affiliations:** ^1^Department of Pediatrics, Graduate School of Medicine, Kobe University, Kobe, Japan; ^2^Faculty of Health Sciences, Kobe Tokiwa University, Kobe, Japan; ^3^Graduate School of Science, Technology and Innovation, Kobe University, Kobe, Japan; ^4^Organization for Research Initiative and Promotion, Tottori University, Yonago, Japan

**Keywords:** Duchenne muscular dystrophy, Becker muscular dystrophy, serum titin, serum creatine kinase, biomarker

## Abstract

**Introduction:**

Duchenne/Becker muscular dystrophies (DMD/BMD) are inherited muscle diseases, collectively referred to as dystrophinopathy, which are characterized by progressive degeneration or loss. Although serum creatine kinase (CK) is a classical biomarker of DMD and BMD, it alone cannot clearly differentiate between severe DMD and milder BMD. Among potential biomarkers, the levels of the fragmented products of titin, a structural muscle protein, may directly reflect the degree of muscle loss in DMD and BMD. Therefore, this study measured the serum titin/creatinine (Cr) ratio and evaluated its discriminatory ability in patients with DMD and BMD.

**Methods:**

The patients with dystrophinopathy and healthy controls were included in this study. Patients were classified by the reading frame rule (out-of-frame, DMD; in-frame, BMD). Exceptional cases in which in-frame variants presented with severe symptoms or out-of-frame variants presented with mild symptoms were considered clinically DMD or clinically BMD, respectively. Serum titin levels were measured using enzyme-linked immunosorbent assay. Serum Cr levels measured on the same day were used to calculate serum titin/Cr ratios.

**Results:**

The DMD, BMD, and control groups included 89 patients (85 DMD and four clinically DMD; aged 3–32 years), 21 patients (16 BMD and five clinically BMD; aged 6–33 years), and five participants (aged 7–12 years), respectively. Although serum CK levels did not differ significantly between DMD and BMD, the serum titin/Cr ratio in DMD was approximately 10 times higher than that in BMD group (*p* < 0.0001). Receiver operating characteristic curve analysis revealed that the ability of serum titin/Cr to distinguish DMD from BMD was superior to that of serum CK. Serum titin/Cr ratios in patients with DMD were higher than those with BMD across all age groups (3–10, 11–15, 16–20, and 21–33), but serum CK levels in patients with DMD were significantly higher than those with BMD only in the 11–15 and 21–33-year age groups.

**Conclusion:**

Unlike serum CK, the serum titin/Cr ratio in patients with DMD was consistently higher than that in patients with BMD, regardless of age. Serum titin/Cr was shown to be a biomarker to discriminate clinical severity in patients with dystrophinopathy.

## 1 Introduction

Duchenne/Becker muscular dystrophies (DMD/BMD), collectively known as dystrophinopathies, are inherited muscle disease caused by an abnormality in the *DMD* gene that encodes the dystrophin protein. The relationship between genotype and phenotype in DMD and BMD can be explained using the reading frame rule (1). Specifically, out-of-frame variants that shift the protein translation reading frame, as determined by codons composed of three bases, lead to dystrophin deficiency and result in a severe phenotype of DMD. In contrast, in-frame variants that preserve the reading frame can produce a truncated form of dystrophin and give rise to the milder BMD phenotype. Patients with DMD or BMD exhibit progressive muscle loss and weakness. In the natural history of DMD, patients typically become non-ambulatory during their teenage years (2). In contrast, disease progression in BMD is more gradual and the age at loss of ambulation varies considerably among patients, with some patients not developing symptoms until adulthood (3).

Titin is a large protein with a molecular weight of approximately 3 million Dalton (4, 5). It spans the Z-disc and M-band of the muscle sarcomere, binds to actin and myosin, and plays a critical role in muscle extensibility and elasticity (5, 6). Titin is specifically degraded by proteases such as trypsin (5). Once titin is degraded, the alignment of myosin and actin is disrupted, leading to significantly reduced active muscle tension (7). Degraded titin fragments have been detected in the urine of patients with DMD and BMD (8–11). The levels of these products are >700- and >30-fold higher in patients with DMD and BMD, respectively, compared with healthy individuals, suggesting that urinary titin may be a biomarker for muscle degeneration (10).

Titin degradation products leak from muscle into the bloodstream and are subsequently excreted in the urine (12). Therefore, the concentration of titin fragments in the blood may directly reflect the degree of muscle degeneration in DMD and BMD. To date, serum titin levels in DMD patients have been assessed using non-quantitative approaches (13, 14); however, no studies have reported precise quantification or comparisons with BMD cases. In this study, we measured the serum titin/creatinine (Cr) ratios in 110 patients with dystrophinopathy and compared them between patients with DMD and BMD with different clinical severities and unaffected controls to evaluate the discriminative ability of this measure. The primary objective was to determine whether the serum titin/Cr ratio could distinguish DMD from BMD more accurately than conventional biomarkers, including serum creatine kinase (CK).

## 2 Material and methods

### 2.1 Participants

This study enrolled male patients with pathogenic *DMD* variants who attended the Pediatric Department of Kobe University Hospital and with residual serum samples between March 1991 and October 2024. Patients who received disease-modifying treatments other than prednisolone before specimen collection were excluded. Using genomic and/or complementary DNA analyses as previously described (15), the effect of each *DMD* variant on the mRNA reading frame was determined and designated as out-of-frame or in-frame. Based on the reading frame rule, patients with out-of-frame variants were classified as DMD and those with in-frame variants as BMD. The patients with nonsense variants were classified as DMD. In addition, since there were known exceptions to the reading frame rule (16), we also evaluated their clinical phenotype. Patients with out-of-frame mutations who remained ambulatory beyond age 15 or showed no significant Gowers sign by age 8 was considered “clinically BMD” (an outlier) and classified as BMD. While patients with in-frame mutations who lost ambulation by age 15 were considered “clinically DMD” (another outlier) and classified as DMD. The control group included pediatric patients who visited our hospital for follow-up regarding short stature or obesity during the same period and with available residual serum samples.

### 2.2 Serum samples and measurement of serum titin and CK levels

Serum samples were obtained from the leftover specimens from routine blood tests and were stored at −20°C until analysis. Serum titin levels were measured using the Human Titin-N Fragment (Serum) enzyme-linked immunosorbent assay (ELISA) kit (Immuno-Biological Laboratories Co. Ltd., Fujioka, Japan) according to the manufacturer's instructions. All samples were assayed in duplicate under blinded conditions, and the mean values were used for analysis.

Serum Cr levels were measured using an enzymatic method using the Signus Auto CR kit (Shino-Test Corporation, Tokyo, Japan), while serum CK levels were measured using the Cygnus Auto CK kit (Shino-Test Corporation, Tokyo, Japan). The serum titin/Cr ratio was calculated by dividing the serum titin concentration by the serum Cr concentration and expressed in pmol/mg Cr. The serum CK/Cr ratio was calculated by dividing the serum CK concentration by the serum Cr concentration and expressed in U/mg Cr.

### 2.3 Statistical analysis

For each patient, one serum titin value was used for analysis. Clinical data for statistical analyses was retrospectively obtained from medical charts. The Shapiro–Wilk test was employed to assess the normality of the dataset, with *p* < 0.05 indicating a non-normal distribution. Student's *t*-test was used for comparisons between two groups if the data were normally distributed; otherwise, the Mann–Whitney *U*-test was used. For comparisons among three or more groups, analysis of variance (ANOVA) or the Kruskal–Wallis test was used, as appropriate. Receiver operating characteristic (ROC) curves and corresponding areas under the curve (AUCs) were calculated to evaluate discriminative performance. Cook's distance was calculated and observations > 4/N were defined as outliers. A correlation coefficient (*r*) was determined using Spearman's rank correlation coefficient; |*r*| < 0.4, 0.4–0.7, and >0.7 were classified as weak, moderate, and strong correlations, respectively. Categorical variables were compared using Fisher's exact test. Statistical significance was set at a two-tailed *p* < 0.05. For multiple testing correction performed using Bonferroni adjustment, *p* < 0.003125 was considered significant. Effect sizes were expressed as Cohen's *d* for two group comparisons. A priori power analysis using Cohen's *d* = 0.8, α = 0.05, and 1–β = 0.80 indicated that 26 participants per group (total *N* = 52) would be required for the primary comparison. As this was a retrospective study, we included all available samples. All statistical analyses were performed using GraphPad Prism version 10.4.0 (GraphPad Software, Boston, USA).

## 3 Results

### 3.1 Participant characteristics

This study included a total of 110 patients. Among them, 85 were DMD and four were classified as clinically DMD, whereas 16 had BMD and five were classified as clinically BMD. Nine cases (8.2%) deviated from the classical genotype–phenotype correlation. Five healthy controls (three males and two females) were also enrolled. All participants were ethnically East Asian. The mean ages at the time of specimen collection (mean ± SD, range) did not differ significantly among the DMD group (DMD and clinically DMD), BMD group (BMD and clinically BMD), and control [16.0 ± 6.5 years (range: 3–32 years) vs. 15.9 ± 7.1 years (6–33 years) vs. 10.0 ± 1.9 years (range: 7–12 years); *p* = 0.10] (Table 1). There was no difference in age at specimen collection between DMD, clinically DMD, BMD, and clinically BMD (*p* = 0.26) (Table 2).

**Table 1:** Clinical characteristics of patients with DMD, BMD, and controls.

***N***	**DMD group**	**BMD group**	**Control**	***P*-value (all three groups)**	***P*-value (DMD vs. BMD groups)**
	**89**	**21**	**5**		
Age (mean ± SD), years	16.0 ± 6.5	15.9 ± 7.1	10 ± 1.9	*p* = 0.10	*p* = 0.95
Ambulatory, *n* (%)	15 (16.9)	18 (85.7)	5 (100)		*p* < 0.0001
Oral prednisone use, *n* (%)	16 (18.0)	2 (9.5)	0 (0)		*p* = 0.52
NPPV use, *n* (%)	18 (20.2)	1 (4.8)	0 (0)		*p* = 0.12

Clinical data from the time of sample collection were used. DMD, Duchenne muscular dystrophy; BMD, Becker muscular dystrophy; NPPV, noninvasive positive pressure ventilation.

**Table 2:** Clinical characteristics of patients with DMD, clinically DMD, BMD and clinically BMD.

***N***	**DMD**	**Clinically DMD**	**BMD**	**Clinically BMD**	**Control**	***P*-value (all five groups)**	***P*-value (DMD vs. BMD)**
	**85**	**4**	**16**	**5**	**5**		
Age (mean ± SD), years	16.1 ± 6.6	12.3 ± 3.4	16.1 ± 6.1	15.1 ± 10.7	10 ± 1.9	*p* = 0.26	*p* = 0.96
Ambulatory, *n* (%)	14 (16.5)	1 (25)	15 (93.8)	3 (60)	5 (100)	*p* < 0.0001	*p* < 0.0001
Oral prednisone use, *n* (%)	15 (17.6)	1 (25)	1 (6.3)	1 (20)	0 (0)	*p* = 0.31	*p* = 0.46
NPPV use, *n* (%)	18 (21.2)	0 (0)	0 (0)	1 (20)	0 (0)	*p* = 0.18	*p* = 0.07

Clinical data from the time of sample collection were used. DMD, Duchenne muscular dystrophy; BMD, Becker muscular dystrophy; NPPV, noninvasive positive pressure ventilation.

Regarding the participants' clinical background at the time of specimen collection, the number of ambulatory patients was significantly lower in the DMD group compared with the BMD group [15 (16.9%) vs. 18 (85.7%); *p* < 0.0001] (Table 1). The rates of oral prednisolone use or noninvasive positive-pressure ventilation (NPPV) did not differ significantly between the groups. Similarly, among the DMD and BMD subgroups, a significant difference was observed only in the rate of loss of ambulation (*p* < 0.0001) (Table 2).

### 3.2 Serum CK, serum CK/Cr ratio, serum titin, and serum titin/Cr ratio in the DMD, BMD, and control groups

**Figure 1:**
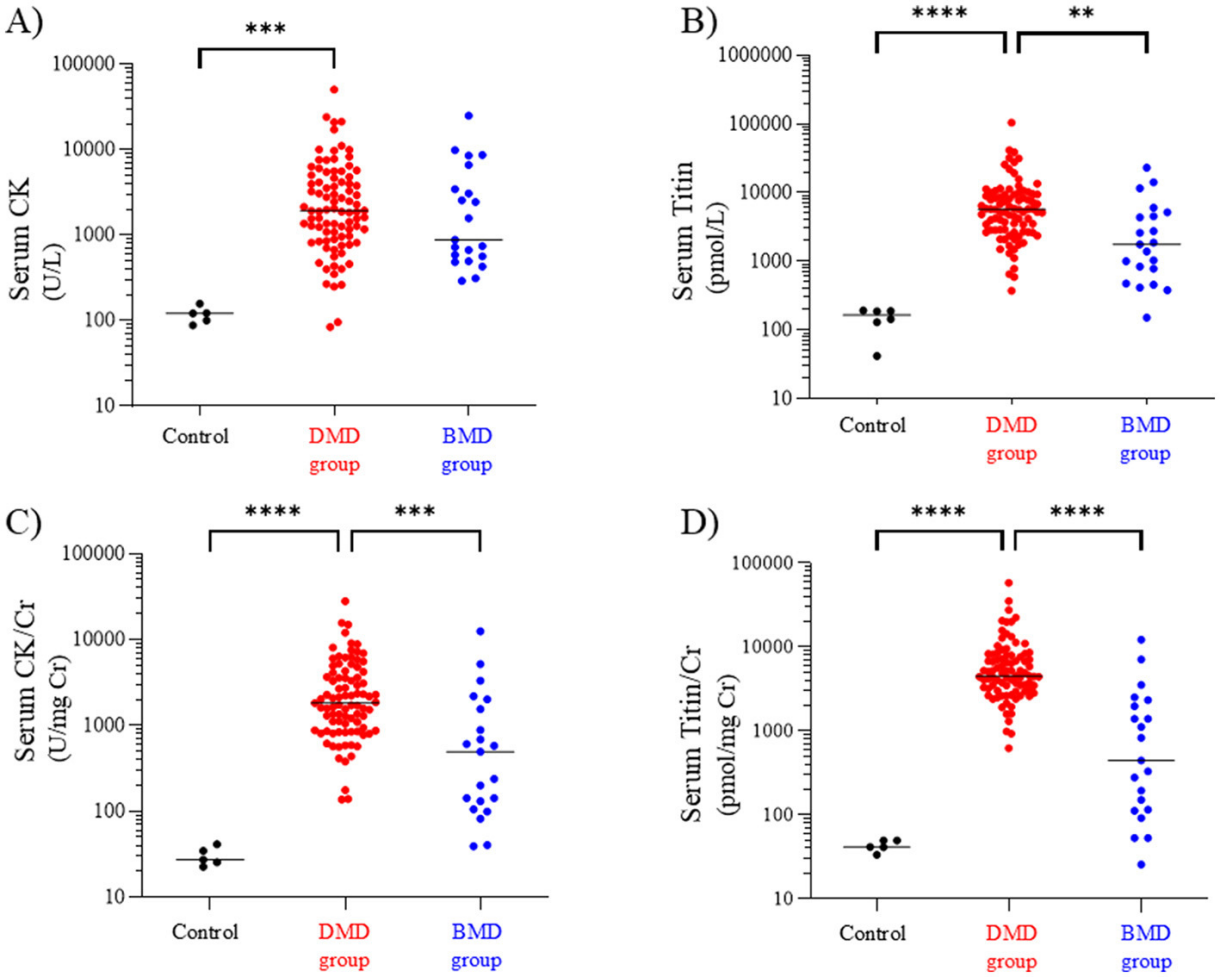
Serum CK **(A)**, titin **(B)**, CK/Cr ratio **(C)**, and titin/Cr ratio **(D)** in the DMD, BMD, and control groups. Black, red, and blue symbols: individual values in the control, DMD, and BMD groups, respectively. **p < 0.01, ***p < 0.001, ****p < 0.0001. CK, creatine kinase; Cr, creatinine; DMD, Duchenne muscular dystrophy; BMD, Becker muscular dystrophy.

The median (range) serum CK values in the DMD group (DMD and clinically DMD), BMD group (BMD and clinically BMD), and control groups were 1,931 (84–50,659), 883 (292–25,006), and 122 (88–158) U/L, respectively. The serum CK level in the DMD group was significantly higher than that in the control group but did not differ significantly from that in the BMD group (Figure 1A) [Cohen's *d* (*d*) = 0.07, 95% confidential interval (CI) −0.41 to 0.54].

In contrast, the median (range) serum titin values in the DMD, BMD, and control groups were 5,704 (374–104,525), 1,768 (151–31,364), and 187 (131–193) pmol/L, respectively, with the DMD group showing a significantly higher serum titin level than the BMD and control groups (*p* < 0.01 and *p* < 0.0001, respectively) (Figure 1B) (*d* = 0.40, 95% CI −0.09 to 0.88).

Next, we compared the Cr-corrected values of the CK and titin levels. The median (range) serum CK/Cr ratios in the DMD, BMD, and control groups were 1,827 (137–28,144), 495 (40–12,503), and 27 (23–42) U/mg Cr, respectively; the DMD group had significantly higher values than the BMD and control groups (*p* < 0.001 and *p* < 0.0001, respectively) (Figure 1C) (*d* = 0.46, 95% CI −0.02 to 0.94). Similarly, the median serum titin/Cr ratios in the DMD, BMD, and control groups were 4,522 (623–58,070), 446 (26–22,403), and 42 (34–50) pmol/mg Cr, respectively, and were significantly higher in the DMD group than in the BMD and control groups (*p* < 0.0001) (Figure 1D). Notably, the median serum CK/Cr ratio in the DMD group was approximately 3.7 times that in the BMD group, whereas the serum titin/Cr ratio in the DMD group was approximately 10-fold higher than that in the BMD group (*d* = 0.74, 95% CI 0.25–1.22).

### 3.3 Serum CK, serum CK/Cr ratio, serum titin, and serum titin/Cr ratio in the DMD, clinically DMD, BMD and clinically BMD

**Figure 2:**
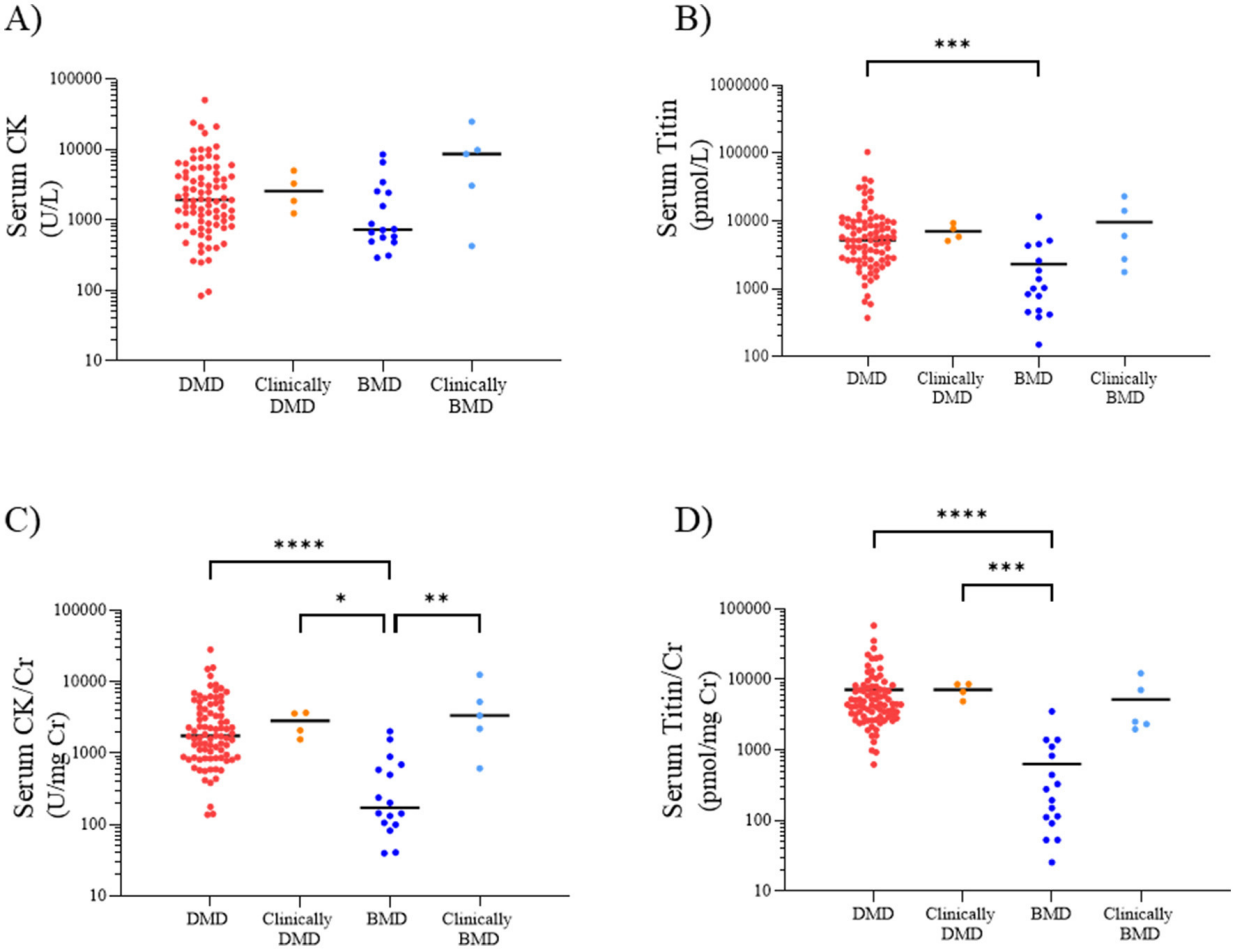
Serum CK **(A)**, titin **(B)**, CK/Cr ratio **(C)**, and titin/Cr ratio **(D)** in the DMD, clinically DMD, BMD, and clinically BMD. Red, orange, blue, and light blue symbols represent individual values for the DMD, clinically DMD, BMD, and clinically BMD, respectively. *p < 0.05, **p < 0.01, ***p < 0.001, ****p < 0.0001. CK, creatine kinase; Cr, creatinine; DMD, Duchenne muscular dystrophy; BMD, Becker muscular dystrophy.

We next compared the serum CK, CK/Cr, titin, and titin/Cr values among the DMD, clinically DMD, BMD, and clinically BMD. The median (range) serum CK values in the DMD, clinically DMD, BMD, and clinically BMD were 1,931 (84–50,659), 2,588 (1,245–5,016), 732 (292–8,547), and 8,661 (428–25,006) U/L, respectively. There was no significant difference in serum CK levels among the four groups (Figure 2A).

In contrast, the median (range) serum titin values in the DMD, clinically DMD, BMD, and clinically BMD were 5,246 (374–104,525), 6,815 (5,111–9,373), 1,021(151–11,653), and 6,064 (1,768–23,214) pmol/L, respectively, with the DMD showing a significantly higher serum titin level than the BMD (*p* < 0.001) (Figure 2B) (*d* = 0.54, 95% CI 0.39–0.90).

The median (range) serum CK/Cr ratios in the DMD, clinically DMD, BMD, and clinically BMD were 1,742 (137–28,144), 2,829 (1,555–3,678), 172 (40–2,011), and 3,331 (611–12,503) U/mg Cr, respectively; the DMD had significantly higher values than the BMD (*p* < 0.001) (Figure 2C) (*d* = 0.74, 95% CI 0.59–1.04). Similarly, the median serum titin/Cr ratios in the DMD, clinically DMD, BMD, and clinically BMD were 4,442 (623–58,070), 7,607 (4,901–8,610), 237 (26–3,531), and 2,525 (1,965–12,218) pmol/mg Cr, respectively, and were significantly higher in the DMD than in the BMD (*p* < 0.001) (Figure 2D) (*d* = 0.86, 95% CI 0.69–1.30). In contrast to the CK/Cr findings, the titin/Cr ratio did not differ significantly between the BMD and clinically BMD.

### 3.4 Discriminative ability between DMD and BMD is highest for serum titin/Cr

**Figure 3:**
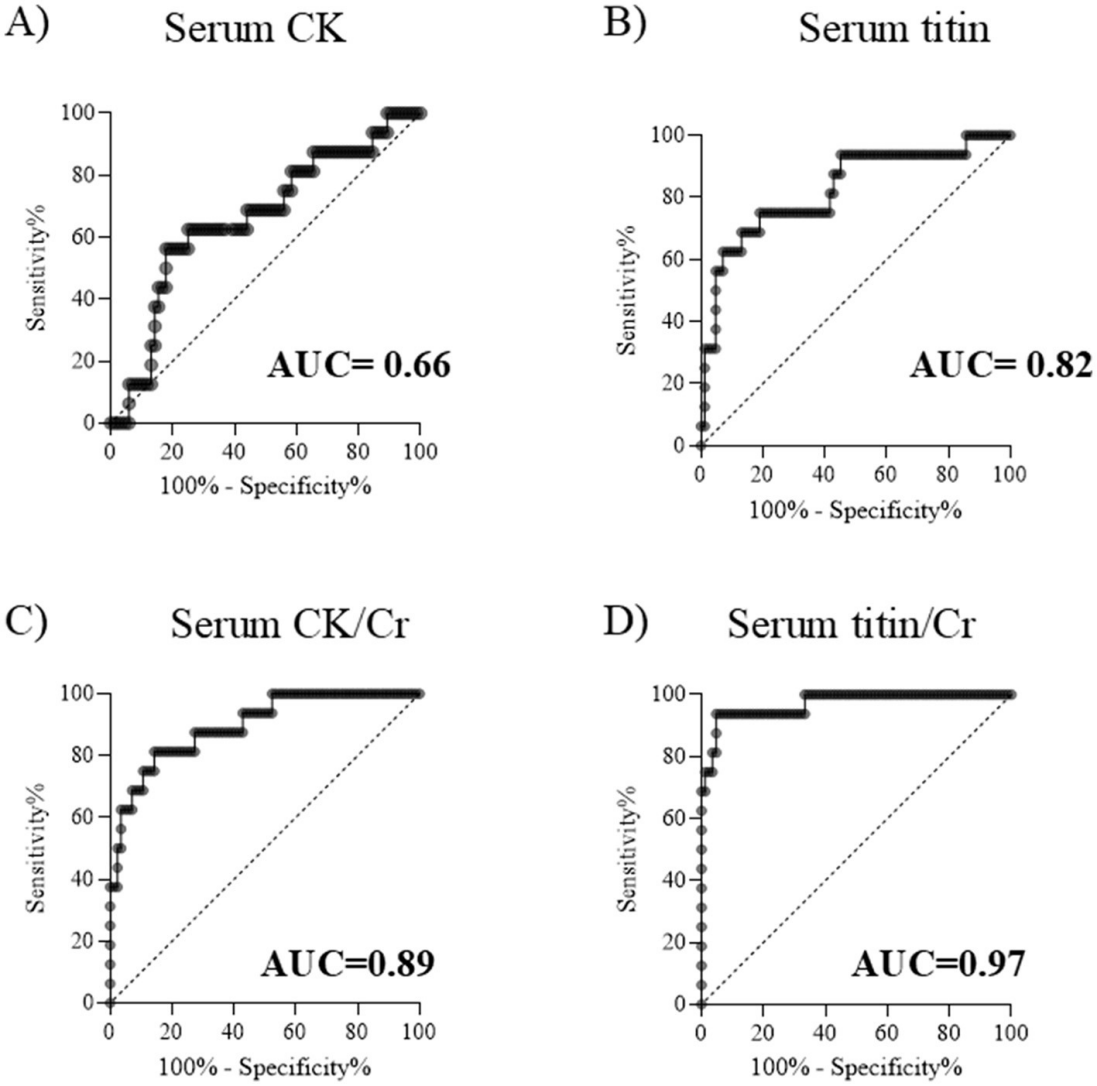
ROC curves for distinguishing DMD from BMD. Serum **(A)** CK, **(B)** titin, **(C)** CK/Cr ratio, and **(D)** titin/Cr ratio. ROC, receiver operating characteristic; DMD, Duchenne muscular dystrophy; BMD, Becker muscular dystrophy; CK, creatine kinase; Cr, creatinine; AUC, area under the curve.

To compare the ability to distinguish between DMD and BMD, receiver operating characteristic (ROC) curves were generated using serum CK, CK/Cr, titin, and titin/Cr values obtained from patients with DMD and BMD, and the area under the curve (AUC) was calculated. An AUC value closer to 1 indicates a better discriminative ability. The AUCs for serum CK and serum titin were 0.66 and 0.82, respectively, indicating that serum titin had superior discriminative performance compared to serum CK (Figures 3A, B). Furthermore, the AUC for serum titin/Cr ratio was greater than that for serum CK/Cr (0.97 vs. 0.89, respectively), demonstrating that serum titin/Cr ratio had better discriminative ability than serum CK/Cr ratio (Figures 3C, D). Among the four markers evaluated, the serum titin/Cr ratio exhibited the highest AUC and the best overall discriminative ability. After excluding outliers (CK: 3 cases; CK/Cr: 8 cases; titin: 8 cases; titin/Cr: 8 cases), the AUCs changed as follows: CK increased from 0.66 to 0.77; CK/Cr from 0.89 to 0.99; titin from 0.82 to 0.98; and titin/Cr from 0.97 to 0.99.

### 3.5 Serum titin/Cr in DMD is higher than in BMD regardless of age

In DMD/BMD patients, age has been shown to affect serum CK levels (17). When examining age-related changes in serum CK, CK/Cr, serum titin, and serum titin/Cr ratios in patients with DMD/BMD, age-related trends were observed in both groups (Supplementary Figures 1A–D). Therefore, to evaluate age-specific differences between DMD group (DMD and clinically DMD) and BMD group (BMD and clinically BMD), we compared serum CK, CK/Cr, titin, and titin/Cr values in the 3–10, 11–15, 16–20, and 21–33-year age groups.

**Figure 4:**
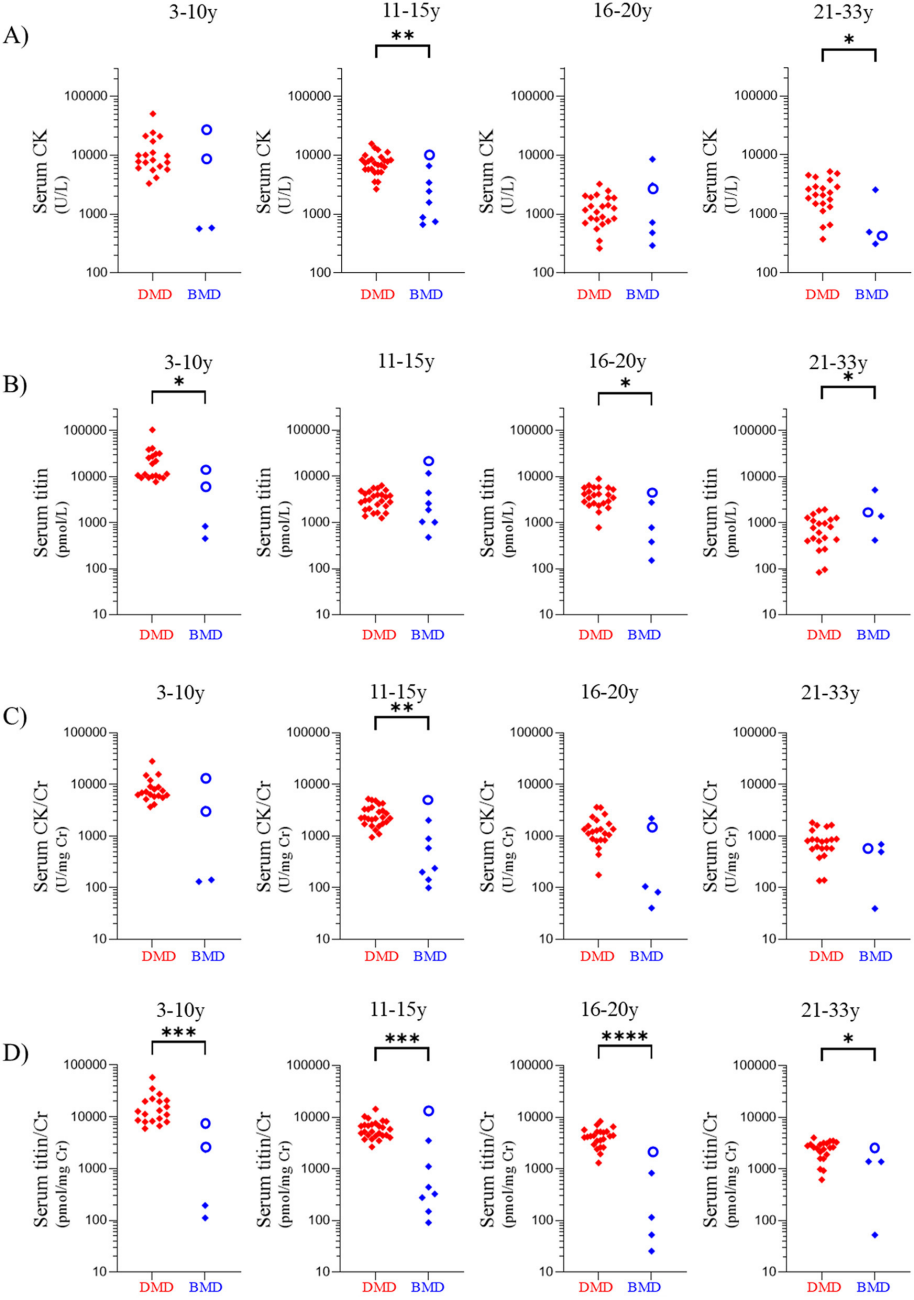
Comparisons of values in patients with DMD group and BMD group according to age group. Age groups: 3–10, 11–15, 16–20, and 21–35 years. Serum **(A)** CK, **(B)** titin, **(C)** CK/Cr ratio, and **(D)** titin/Cr ratio. Red and blue symbols: individual values for patients with DMD group and BMD group, respectively. Open circles indicate the value of clinically BMD. *p < 0.05, **p < 0.01, ***p < 0.001, ****p < 0.0001. The statistical results are those obtained by the Mann-Whitney *U*-test before applying the Bonferroni correction. DMD, Duchenne muscular dystrophy; BMD, Becker muscular dystrophy; CK, creatine kinase; Cr, creatinine.

Serum CK levels in the DMD group were significantly higher than those in the BMD group in the 11–15- and 21–33-year age groups (*p* < 0.01 and *p* < 0.05, respectively) (Figure 4A) (Cohen's d values are listed in Supplementary Table 1). Serum titin levels in patients with DMD were significantly higher than those in patients with BMD in the 3–10, 16–20, and 21–33-year age groups (*p* < 0.05) (Figure 4B), whereas the 11–15-year age group showed no significant difference between patients with DMD and those with BMD (*p* = 0.51). Similarly, the serum CK/Cr ratio in patients with DMD was significantly higher than that in patients with BMD only in the 11–15-year age group (*p* < 0.01) (Figure 4C). In contrast, the serum titin/Cr ratio was significantly higher in patients with DMD than in those with BMD group across all age groups (Figure 4D). Following Bonferroni correction, serum titin/Cr levels remained statistically significant in the 3–10, 11–15, and 16–20 year age groups.

Among the patients with BMD group, seven exhibited serum titin/Cr ratios comparable to those observed in patients with DMD group (Figure 4D). Of these seven patients, three were BMD and four were clinically BMD. Three of seven (42.9%) were non-ambulatory (Supplementary Table 2). In contrast, none of the remaining 14 patients with BMD group were non-ambulatory, indicating that high serum titin/Cr ratios were significantly associated with non-ambulation (*p* < 0.05).

### 3.6 Ambulatory status in DMD patients affects serum CK/Cr but not serum titin/Cr ratio

As serum CK levels increase with physical activity (16), the serum titin/Cr ratio may be similarly influenced by exercise. Therefore, we compared serum CK/Cr and serum titin/Cr ratios between ambulatory and non-ambulatory patients with DMD group (DMD and clinically DMD) aged 3–10 and 11–15 years.

**Figure 5:**
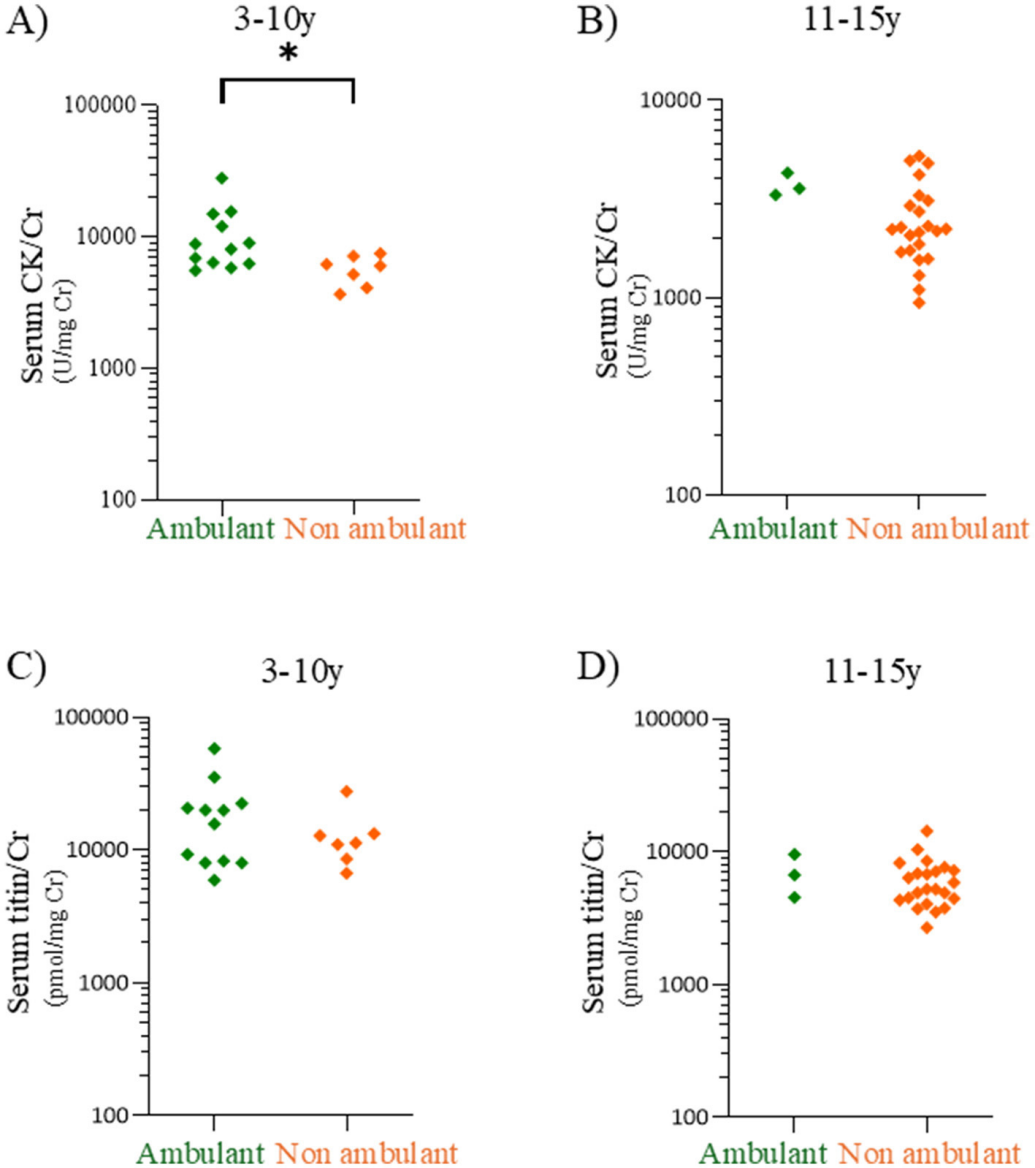
Comparisons of serum CK/Cr and serum titin/Cr between ambulatory and non-ambulatory patients with DMD patients. Serum CK/Cr ratios in the 3–10-year **(A)** and 11–15-year age **(B)** age groups. Serum titin/Cr ratios in the 3–10-year **(C)** and 11–15-year **(D)** age groups. Green and orange dots: individual values for ambulatory and non-ambulatory patients with DMD, respectively. CK, creatine kinase; Cr, creatinine; DMD, Duchenne muscular dystrophy. *p < 0.05.

Serum CK/Cr and titin/Cr ratios were compared between ambulatory and non-ambulatory patients with DMD group in the 3–10- and 11–15-year age groups (Figures 5A–D). In the 3–10-year age group, the median CK/Cr ratio of ambulatory patients was significantly higher than that of non-ambulatory patients (8,486 vs. 6,050 U/mg Cr, *p* < 0.05) (Figure 5A) (Cohen's *d* values are listed in Supplementary Table 3). In contrast, the median serum titin/Cr values did not differ significantly between ambulatory and non-ambulatory patients in either the 3–10-year or 11–15-year age group (*p* = 0.59 and *p* = 0.54, respectively) (Figures 5C, D).

### 3.7 Serum titin/Cr levels and renal and cardiac function

Serum cystatin C, blood urea nitrogen and estimated glomerular filtration rate, as indicators of renal function, were not strongly correlated with serum titin/Cr levels in either DMD or BMD patients (Supplementary Table 4). Similarly, the cardiac function indicators plasma brain natriuretic peptide and left ventricular ejection fraction measured by echocardiography also demonstrated no strong correlation with serum titin/Cr levels in the same groups.

## 4 Discussion

Titin is present in the serum of patients with DMD (13, 14). However, proteomic analyses have not previously detected titin in the serum of healthy subjects (13, 14). Additionally, no studies have investigated serum titin in patients with BMD. In pediatric patients with DMD aged 4–15 years, serum titin levels were shown to decrease with age (14). However, because serum CK levels exhibit similar age-related changes in that analysis, the clinical significance of serum titin levels remains unclear. This study is the first to quantitatively measure serum titin levels using an ELISA method in 89 DMD patients, 21 BMD patients, and 5 healthy children. The results demonstrated that serum titin levels in DMD patients were significantly higher than those in BMD patients and healthy controls. Further, serum titin/Cr has been shown to be a marker with better discriminative ability than CK between DMD and BMD.

Because muscle atrophy progresses with age in DMD (19), low serum titin levels may be difficult to interpret; specifically, it becomes challenging to determine whether they reflect a reduction in muscle mass or a decrease in titin degradation. To address this, we evaluated serum titin/Cr ratios. Cr is a metabolic product produced at a constant rate from the degradation of muscle creatine in proportion to the muscle mass. Therefore, serum Cr concentration reflects skeletal muscle mass (20). Thus, the serum titin/Cr ratio represents the amount of titin degradation per unit of skeletal muscle mass. Indeed, although the serum titin level was significantly lower in patients with advanced DMD aged > 20 years (with reduced muscle mass) than in age-matched patients with BMD in the present study (Figure 4B), the serum titin/Cr ratio was significantly higher in patients with DMD (Figure 4D). The serum titin/Cr ratio showed no strong correlation with clinical indicators of renal or cardiac function in either DMD or BMD patients (Supplementary Table 1). This suggests that serum titin/Cr levels were not substantially influenced by renal excretion and that the measured titin is likely derived primarily from skeletal muscle rather than myocardium. However, it should be noted that while urine titin levels have been reported to correlate with cardiac damage in BMD (10) and to be associated with mortality in patients with heart failure unrelated to muscular dystrophy (21), no studies have investigated the association between serum titin levels and heart failure, either in patients with DMD/BMD or in other populations. These results suggested increased titin degradation in the skeletal muscles of patients with DMD than in those with BMD. Active titin degradation may be associated with the severity of the clinical phenotype of *DMD* abnormalities, indicating that the serum titin/Cr ratio may be a useful marker for differentiating between DMD and BMD.

In the ROC analysis using raw data, serum titin/Cr showed the highest AUC for distinguishing between DMD and BMD. After excluding outliers, the AUCs of titin/Cr and creatine CK/Cr became comparable. While the AUC for titin/Cr remained stable regardless of outlier inclusion, that of CK/Cr increased notably when outliers were removed, suggesting prior underestimation. However, outliers may reflect clinically meaningful variability, and their exclusion could bias results (22) or misrepresent the patient population. In view of all these findings, titin/Cr was considered a highly reliable biomarker, as its AUC remained close to 1.0 regardless of outlier exclusion

Although DMD/BMD are generally classified based on the reading frame rule—with out-of-frame mutations associated with a DMD genotype and in-frame mutations with a BMD genotype—exceptions occur in 4–9% of cases due to factors such as alternative splicing, modifier genes, and other molecular mechanisms (16). In our study of 110 patients, nine cases (8.2%) deviated from the classical genotype–phenotype correlation, consistent with previous reports. It was interesting that clinically BMD, which is an outlier, showed different serum CK/Cr and serum titin/Cr values than BMD (Figures 2C, D). When comparing the BMD and clinically BMD groups, the CK/Cr ratio was significantly higher in the clinically BMD group (*p* < 0.01). In contrast, the titin/Cr ratio did not differ significantly between these two groups. However, in analyses of different age groups, clinically BMD always showed the highest serum titin/Cr value, unlike serum CK/Cr (Figure 4D). This suggests that serum titin/Cr may more sensitively represent the severity of the genotype than serum CK. However, considerable overlap in serum CK/Cr and titin/Cr levels was observed between the clinically BMD and DMD groups. Therefore, it should be recognized that serum titin/Cr may not provide superior discriminatory power compared to serum CK/Cr when assessing differences in disease severity between these two clinical groups. Due to the limited sample size of this study, it was not possible to conduct a statistical analysis considering age, therefore further research is needed.

Serum CK, a classical biomarker of DMD, is commonly used to diagnose DMD (23) and is associated with functional motor scores (24). However, serum CK levels are affected by factors such as muscle trauma and exercise (18). In DMD and BMD, serum CK values exhibit considerable variability and overlap, even among patients of the same age (17). Moreover, the rate of age-related decline in serum CK is higher in DMD than in BMD, resulting in higher serum CK levels in younger patients with DMD compared with those with BMD but lower levels in advanced stages compared to age-matched patients with BMD (17). Therefore, serum CK has not been effectively utilized as a marker to differentiate between the varying severities of DMD and BMD.

In the present study, serum CK levels did not differ significantly between DMD and BMD groups in the 3–10-year and 16–20-year age groups (Figure 4A). Moreover, its discriminative ability in the ROC analysis was suboptimal (Figure 3A). In contrast, the serum titin/Cr ratio was significantly higher in DMD than in BMD across all patient age groups, including younger patients to those aged ≥ 21 years advanced stage (Figure 4D). Even after adjusting for multiple comparisons, the titin/Cr ratio remained significantly different in the pediatric population under 20 years of age. These findings suggest that the serum titin/Cr ratio in pediatric patients overcomes the limitations posed by age-related changes effects on serum CK levels, thereby emerging as a novel and superior biomarker for differentiating between pediatric DMD and BMD. Furthermore, unlike serum CK levels, serum titin/Cr ratios did not differ significantly between ambulatory and non-ambulatory pediatric patients (Figures 5C, D), suggesting that his measure may serve as a biomarker independent of exercise.

Titin in the blood is attracting attention as a potential therapeutic marker. In DMD model mice that received the full-length human *DMD* replacement therapy, plasma titin was suggested to be more sensitive marker than plasma CK (25) In recent years, several active clinical trials for novel treatments for DMD such as exon-skipping therapy and micro-dystrophin therapy, have been conducted with some of these approaches already approved in certain countries (26). Exon-skipping therapy involves the removal of certain exons from mRNA during splicing using antisense oligonucleotides to make an out-of-frame variants into an in-frame variant that can produce truncated dystrophin protein (27). In contrast, micro-dystrophin therapy utilizes viral vector products carrying *DMD* cDNA encoding a truncated dystrophin protein (28). Both treatments aim to enable patients with DMD who lack full-length dystrophin, to express a truncated yet functional dystrophin protein similar to that found in patients with BMD, effectively shifting the DMD phenotype toward the BMD-like phenotype.

Traditionally, DMD-focused clinical trials have used motor function assessment as the primary endpoint (29). However, variability in patients' motor function trajectories (30) makes it challenging to evaluate treatment efficacy especially within the limited duration of clinical trials. Given the current need for reliable and objective biomarkers to assess therapeutic efficacy in DMD, the potential of serum titin should be explored through investigation through prospective longitudinal studies.

This study has some limitations. First, this was a cross-sectional study in which samples were collected from patients at arbitrary time points. Second, the sample size and age range of the participants were limited. In particular, the number of patients with BMD is below the number estimated by priori power analysis. The life expectancy of patients with DMD has improved, with some patients living > 40 years of age (31). In patients with BMD, considerable number of individuals are in their 70s (32). A large-scale cohort study including patients from younger to advanced patients is required to confirm the discriminative ability of serum titin/Cr ratios across all disease stages. Third, this study examined the association between serum titin levels and selected cardiac and renal function markers; however, other relevant clinical parameters were not assessed. Further comprehensive studies are needed to identify potential confounders affecting serum titin levels. Finally, the impact of long-term storage on measured values remains uncertain. In a preliminary analysis, serum titin levels after 7, and 14 days of storage were 78.0 and 80.1% of baseline, respectively. As all samples in this study were stored for over 2 weeks, storage duration may have influenced the results.

## 5 Conclusion

Unlike serum CK, serum titin/Cr ratios in pediatric patients with DMD were consistently higher than those in pediatric patients with BMD.

The degree of titin degradation, as indicated by the serum titin/Cr ratio, was associated with the severity of phenotype. Overall, serum itin/Cr was shown to be a biomarker to discriminate severity in patients with dystrophinopathy.

## Data Availability

The raw data supporting the conclusions of this article will be made available by the authors, without undue reservation.
