# Supplementary material for: Serum titin/creatinine ratio as a biomarker for discriminating disease severity in Duchenne and Becker muscular dystrophies
Source: Front Neurol. 2025 Jul 9;16:1591748. doi: 10.3389/fneur.2025.1591748 (PMC12283318; doi:10.3389/fneur.2025.1591748)
Supplement: Supplementary file 1 [file Supplementary_file_1.docx]

Supplementary Material

# Supplementary Figures and Tables

## Supplementary Figure 1


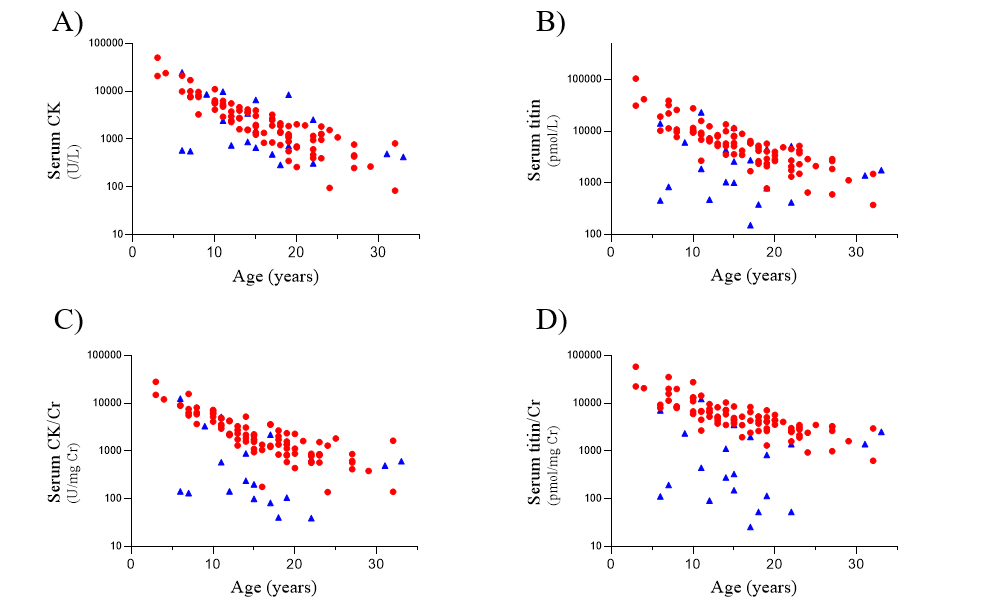


**Supplementary Figure 1.** Correlations between age and (A) serum CK, (B) serum titin, (C) serum CK/Cr ratio, and (D) serum titin/Cr ratio. Red circles and blue triangles: values for patients with DMD and BMD groups, respectively. CK, creatine kinase; Cr, creatinine; DMD, Duchenne muscular dystrophy; BMD, Becker muscular dystrophy

## Supplementary Tables

**Supplementary Table 1.** Effect sizes (Cohen’s d) with 95% confidence intervals for age-stratified comparisons of serum CK, CK/Cr, titin, and titin/Cr between DMD and BMD

|  | Age Group | Cohen’s d | 95% confidence interval |
| --- | --- | --- | --- |
| Serum CK | 3–10 | 0.34 | -0.74–1.43 |
|  | 11–15 | 0.30 | -0.52–1.11 |
|  | 16–20 | 0.03 | -0.94–1.00 |
|  | 21–33 | 0.04 | -0.86–0.95 |
| Serum titin | 3–10 | 0.86 | -0.25–1.96 |
|  | 11–15 | 0.41 | -0.39–1.21 |
|  | 16–20 | 0.21 | -0.77–1.19 |
|  | 21–33 | 0.12 | -0.81–1.04 |
| Serum CK/Cr | 3–10 | 0.84 | -0.27–1.95 |
|  | 11–15 | 0.48 | -0.34–1.31 |
|  | 16–20 | 0.38 | -0.60–1.36 |
|  | 21–33 | 0.32 | -0.58–1.23 |
| Serum titin/Cr | 3–10 | 1.23 | 0.10–2.37 |
|  | 11–15 | 0.67 | -0.15–1.49 |
|  | 16–20 | 0.64 | -0.34–1.62 |
|  | 21–33 | 0.51 | -0.50–1.52 |

CK, creatine kinase; Cr, creatinine; DMD, Duchenne muscular dystrophy; BMD, Becker muscular dystrophy

**Supplementary Table 2.** Clinical characteristics of patients with Becker muscular dystrophy group showing serum titin/Cr level comparable to those of patients with Duchenne muscular dystrophy group

| ID | Age at sampling  (years) | Age at last visit  (years) | *DMD* variant  (Reading frame) | Serum CK  (U/L) | Age at loss of ambulation  (years) | Oral PSL　use | Muscle symptoms |
| --- | --- | --- | --- | --- | --- | --- | --- |
| 1256 | 6 | 10 | Deletion of exons 3–7  Out-of-frame | 25,006 | Not yet reached | No | Gowers’ sign |
| 965 | 11 | 16 | c.10509-10510del  Out-of-frame | 9,878 | Not yet reached | No | Gowers’ sign  Use of stair handrail |
| 1376 | 15 | 17 | Deletion of exons 3–4  In-frame | 6,637 | Not yet reached | No | Difficulty standing up  Toe waking |
| 302 | 17 | 22 | Deletion of exons 3–7  Out-of-frame | 3,086 | 16 | No | Difficulty standing up |
| 1206 | 22 | 23 | Deletion of exons 10–12  In-frame | 2,556 | Not yet reached | No | Gowers’ sign  Difficulty climbing stairs  Toe waking |
| 37 | 31 | 33 | Deletion of exon 3  In-frame | 495 | 16 | No | Difficulty standing up  Difficulty climbing stairs |
| 565 | 33 | 35 | Deletion of exon 45  Out-of-frame | 428 | 18 | No | Difficulty climbing stairs  Gowers’ sign |

*DMD,* dystrophin gene*;* CK, creatine kinase; PSL, prednisolone

Note: The muscle symptoms in this table include those observed at any time during the disease course and not only at the final visit.

**Supplementary Table 3.** Effect sizes (Cohen’s d) with 95% confidence intervals for comparisons of serum CK/Cr and titin/Cr between ambulant and non-ambulant patients with DMD

|  | Age Group | Cohen’s d | 95% confidence interval |
| --- | --- | --- | --- |
| Serum CK/Cr | 3–10 | 0.93 | -0.04–1.91 |
|  | 11–15 | 1.01 | -0.22–2.25 |
| Serum titin/Cr | 3–10 | 0.49 | -0.45–1.44 |
|  | 11–15 | 0.32 | -0.88–1.52 |

CK, creatine kinase; Cr, creatinine; DMD, Duchenne muscular dystrophy; BMD, Becker muscular dystrophy

**Supplementary Table 4.** Correlation between serum titin/Cr and indicators of renal and cardiac function.

|  | Serum titin/Cr | | | |
| --- | --- | --- | --- | --- |
|  | DMD | | BMD | |
|  | N | r | N | r |
| Renal function |  |  |  |  |
| Cystatin C, mg/dL | 89 | 0.18 | 21 | -0.04 |
| BUN, mg/dL | 89 | 0.02 | 21 | -0.20 |
| eGFR *, mL/min/1.73 m² | 89 | 0 | 21 | -0.01 |
| Cardiac function |  |  |  |  |
| Plasma BNP | 87 | -0.14 | 21 | 0.42 |
| LVEF, % | 88 | 0.49 | 19 | -0.29 |

BUN, blood urea nitrogen; BNP, brain natriuretic peptide; eGFR, estimated glomerular filtration rate; LVEF, left ventricular ejection fraction. * eGFR was calculated from serum cystatin C using the equation eGFR (104.1/cystatin C [mg/L] -7.8) for patients with aged <18 years and 133 ×min(CysC/0.8, 1)^–0.499^ × max(CysC/0.8, 1)^–1.328^ × 0.996^Age^ for those aged ≥18 years.
